# Sustained exposure to *Helicobacter pylori* induces immune tolerance by desensitizing TLR6

**DOI:** 10.1007/s10120-023-01461-7

**Published:** 2024-02-04

**Authors:** Xiulin Zhang, Yang He, Xiaolu Zhang, Bo Fu, Zidai Song, Liang Wang, Rui Fu, Xuancheng Lu, Jin Xing, Jianyi Lv, Meng Guo, Xueyun Huo, Xin Liu, Jing Lu, Xiaoyan Du, Zhongming Ge, Zhenwen Chen, Changlong Li

**Affiliations:** 1 Department of Medical Genetics and Developmental Biology, School of Basic Medical Science, Beijing Key Laboratory of Cancer Invasion & Metastasis Research, Laboratory for Clinical Medicine, Capital Medical University, Beijing, People’s Republic of China; 2 State Key Laboratory of Cardiovascular Disease, Fuwai Hospital, Chinese Academy of Medical Science, Beijing, People’s Republic of China; 3 School of Nursing, Dalian Medical University, Dalian, People’s Republic of China; 4 Peking University Ninth School of Clinical Medicine, Beijing, People’s Republic of China; 5 Institute for Laboratory Animal Resources, National Institutes for Food and Drug Control, Beijing, People’s Republic of China; 6 Laboratory Animal Center, Chinese Center for Disease Control and Prevention, Beijing, People’s Republic of China; 7 Division of Comparative Medicine, Massachusetts Institute of Technology, Cambridge, USA

**Keywords:** *H. pylori*, Immune tolerance, TLR6, GES-1 cells, Mongolian gerbils

## Abstract

*Helicobacter pylori* (*H. pylori, Hp*) has been designated a class I carcinogen and is closely associated with severe gastric diseases. During colonization in the gastric mucosa, *H. pylori* develops immune escape by inducing host immune tolerance. The gastric epithelium acts as the first line of defense against *H. pylori*, with Toll-like receptors (TLRs) in gastric epithelial cells being sensitive to *H. pylori* components and subsequently activating the innate immune system. However, the mechanism of immune tolerance induced by *H. pylori* through the TLR signalling pathway has not been fully elucidated. In this research, we detected the expression of TLRs and inflammatory cytokines in GES-1 cells upon sustained exposure to *H. pylori* or *H. pylori* lysate from 1 to 30 generations and in Mongolian gerbils infected with *H. pylori* for 5 to 90 weeks. We found that the levels of TLR6 and inflammatory cytokines first increased and then dropped during the course of *H. pylori* treatment in vitro and in vivo. The restoration of TLR6 potentiated the expression of IL-1β and IL-8 in GES-1 cells, which recruited neutrophils and reduced the colonization of *H. pylori* in the gastric mucosa of gerbils. Mechanistically, we found that persistent infection with *H. pylori* reduces the sensitivity of TLR6 to bacterial components and regulates the expression of inflammatory cytokines in GES-1 cells through TLR6/JNK signaling. The TLR6 agonist obviously alleviated inflammation in vitro and in vivo. Promising results suggest that TLR6 may be a potential candidate immunotherapy drug for *H. pylori* infection.

**Supplementary Information:**

The online version contains supplementary material available at 10.1007/s10120-023-01461-7.

## Introduction

*Helicobacter pylori* (*H. pylori*, *Hp*) is a gram-negative bacterium that has been designated as a class I carcinogen [1]. *H. pylori* infection is closely associated with gastric diseases, including gastritis, peptic ulcer, intestinal metaplasia and adenocarcinoma [2–4]. Upon colonization in the gastric epithelium, *H. pylori* triggers the production of inflammatory cytokines, including interleukin-1β (IL-1β), interleukin-6 (IL-6), interleukin-8 (IL-8) and TNF-alpha [5–8]. Prolonged exposure to these cytokines can lead to cell proliferation and an increased risk of DNA replication errors, thereby promoting tumorigenesis by inhibiting cell autophagy and apoptosis [9–11]. Both experimental and clinical studies have confirmed that *H. pylori* infection and chronic inflammation play crucial roles in the development of gastric cancer [12].

In *H. pylori*-infected individuals, gastric epithelial cells form the initial innate immune defense barrier [13]. Toll-like receptors (TLRs) in gastric epithelial cells are among the earliest determinants of immune activation and are sensitive to bacterial components in the microenvironment [14]. They can sensitize lipopolysaccharide (LPS), flagellin, bacterial lipoproteins, and other microbial cell components [15]. It is plausible that individuals exhibit a strong immune response to *H. pylori* infection through TLR pathways, which is conducive to eliminating the bacteria and inhibiting long-term colonization [16–19]. However, *H. pylori* infection is a long-term process that leads to permanent colonization if not effectively treated [20]. During this process, *H. pylori* can evade the immune system by reducing the inflammatory response and interfering with innate and adaptive immune responses [21]. Research has shown that stromal factors in the gastric mucosa suppress the activation of dendritic cells (DCs) against H. pylori [22, 23]. Additionally, *H. pylori* promotes the production of interleukin-33 (IL-33) by epithelial cells, which may protect against excessive inflammation and support bacterial persistence [7, 24]. Owyang et al. uncovered the ability of *H. pylori* genomic DNA to downregulate inflammatory responses through the TLR9 signaling pathway [19]. Overall, *H. pylori* achieves long-term colonization mainly by evading the immune system through immune tolerance and immune escape [23, 25]. TLRs play a crucial role in immune tolerance induced by microbial pathogens, but the specific mechanism of immune tolerance induced by H. pylori through the TLR signaling pathway has not been fully elucidated [26].

To elucidate the pathogenic mechanism induced by *H. pylori,* researchers typically stimulate gastric epithelial cells with bacteria for up to 72 h in vitro [17, 27–29]. Few studies have described the effects of prolonged stimulation of *H. pylori* on gastric cells,, which is more similar to the actual pathogenesis process of *H. pylori* infection in vivo. In addition, during the proliferation and metabolic processes of colonized bacteria, *H. pylori* are lysed and release a large number of harmful substances [30–33]. Our previous study confirmed that *H. pylori* lysate acts as an "accomplice" to carcinogenesis in the process of *H. pylori*-induced gastric lesions [34]. Thus, our objective was to investigate the immune response of GES-1 cells to prolonged stimulation with *H. pylori* and *H. pylori* lysate. We employed *H. pylori* strain ATCC 43504 (CagA^+^/VacA^+^), initially identified in a gastric cancer patient, which has been used in previous pathogenicity investigations involving GES-1 cell lines exposed to live cells or cell lysates for 30 generations [35, 36]. Subsequently, we examined the expression of TLRs and inflammatory cytokines in cells and *H. pylori*-positive Mongolian gerbils. Our findings revealed that sustained exposure to *H. pylori* or *H. pylori* lysate attenuates the sensitivity of GES-1 cells to TLR6, thereby suppressing the host immune response and facilitating *H. pylori* immune evasion.

## Results

### Sustained exposure to *H. pylori* or *H. pylori* lysate in GES-1 cells inhibited TLR6 expression

**Fig. 1:**
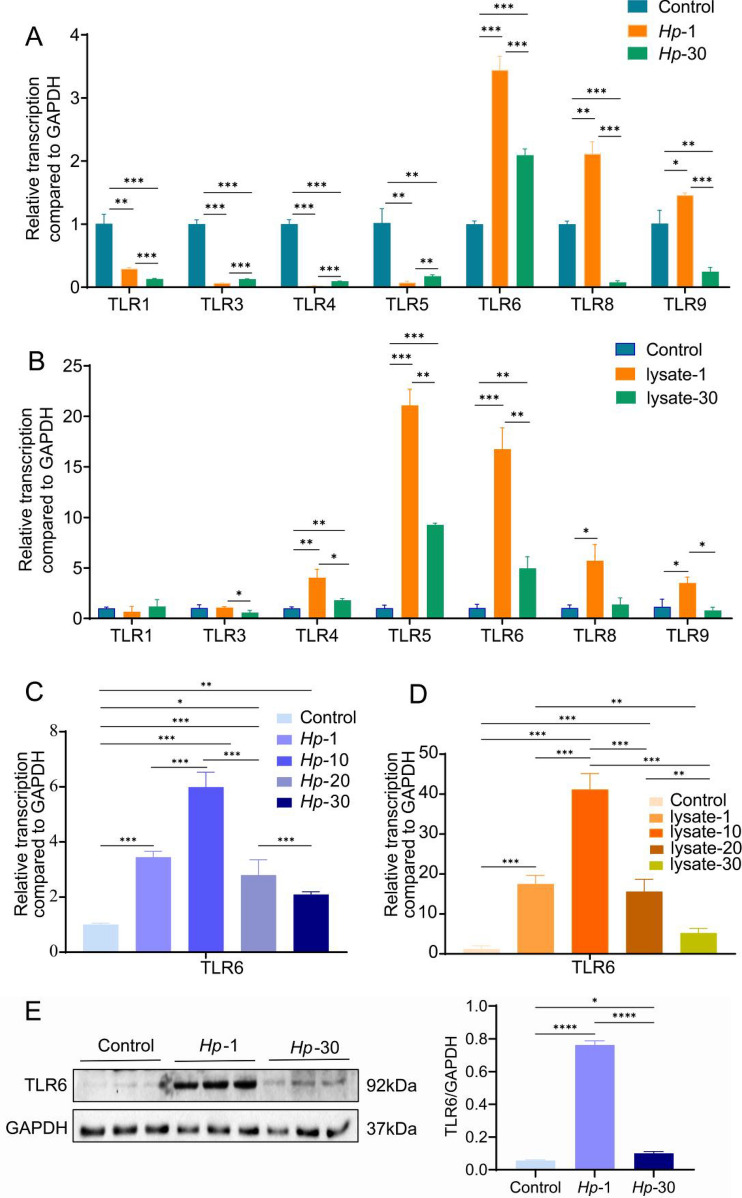

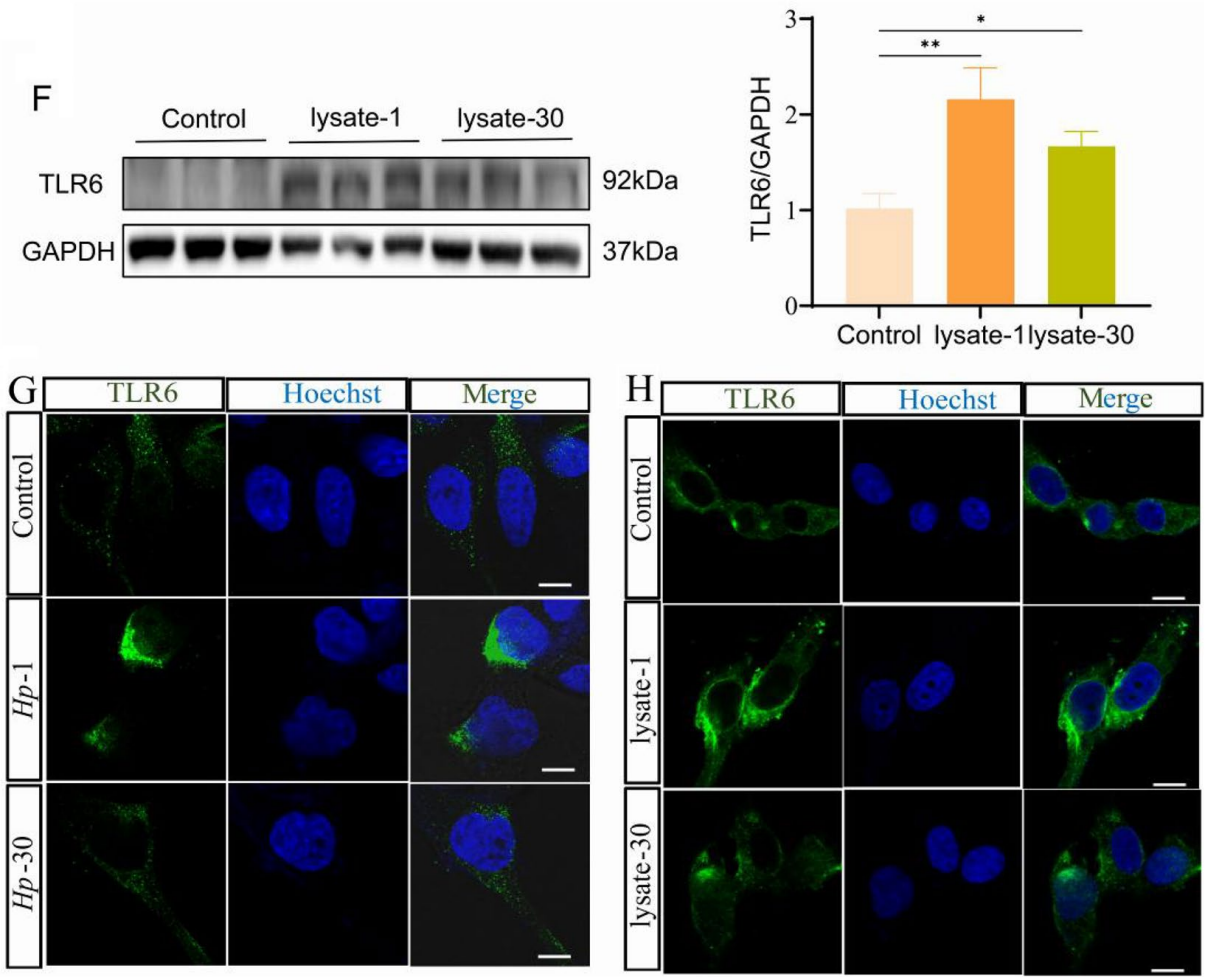
TLRs mRNA expression in (**A**) wild-type GES-1 cells (control), GES-1 cells persistently infected with *H. pylori* for 1 generation (*Hp*-1) and 30 generations (*Hp*-30) or in (**B**) control cells, GES-1 cells co-cultured with *H. pylori* lysate for 1 generation (lysate-1) and 30 generations (lysate-30). TLR6 mRNA expression in (**C**) control cells, *Hp-*1 cells, *Hp-*10 cells, *Hp-*20 cells and *Hp-*30 cells or in (**D**) control cells, lysate*-*1 cells, lysate*-*10 cells, lysate*-*20 cells and lysate*-*30 cells. TLR6 protein expression (**E**) and TLR6 immunofluorescent staining (**G**) in control cells, *Hp-*1 cells and *Hp-*30 cells. TLR6 protein expression (**F**) and TLR6 immunofluorescent staining (**H**) in control cells, lysate*-*1 cells and lysate*-*30 cells. All experiment were performed in triplicate and all data represents means ± SEM. ***P < 0.001, **P < 0.01, *P < 0.05. Scale bars, 10 µm (**G**, **H**)

Inflammatory cytokines may be produced as a result of TLR upregulation during *H. pylori* infection, which contribute to the development of gastric disease [16]. Uncertainty exists regarding the impact of prolonged exposure to *H. pylori* or *H. pylori* lysate on TLR expression in GES-1 cells. Hence, we first examined the mRNA levels of TLR1, TLR2, TLR3, TLR4, TLR5, TLR6, TLR8 and TLR9 in GES-1 cells between wild-type GES-1 cells (control) and GES-1 cells that had been infected with *H. pylori* for one generation (24 h) or for 30 generations (*Hp*-30 cells). TLR2 expression was shown to be extremely low in gastric epithelial cells, which is consistent with previous reports [37]. TLR7, which primarily identifies viral mRNA, has not been demonstrated to be involved in the innate immune response linked to *H. pylori* [26]. Figure 1A demonstrates that compared to wild-type GES-1 cells and *Hp*-30 cells, the mRNA levels of TLR6, TLR8 and TLR9 in *Hp*-1 cells were significantly higher. Then, wild-type GES-1 cells, GES-1 cells exposed to *H. pylori* lysate for 24 h (lysate-1) and GES-1 cells exposed to *H. pylori* lysate for 30 generations (lysate-30) were examined for their mRNA levels of TLRs. Lysate-1 cells displayed significantly increased levels of TLR4, TLR5, TLR6, TLR8 and TLR9 mRNA expression compared to wild-type cells and lysate-30 cells (Fig. 1B). Among them, TLR8 and TLR9 are expressed within intracellular vesicles and mainly recognize RNA or single-stranded DNA of pathogens [38, 39]. TLR6 was dramatically downregulated following prolonged exposure to *H. pylori* or *H. pylori lysate* and showed a significant response to both *H. pylori* and *H. pylori* lysate. As a result, we focused on TLR6 for further characterization in detail.

We then examined TLR6 mRNA levels in wild-type GES-1 cells, *Hp*-1 cells, GES-1 cells that had been infected with *H. pylori* for 10 (*Hp*-10), 20 (*Hp*-20) or 30 (*Hp-*30) generations. The level of TLR6 mRNA peaked at generation 10, then gradually declined from generation 10 to generation 30 (Fig. 1C). The similar pattern was observed in lysate-1 cells, lysate-10, lysate-20 and lysate-30 cells (Fig. 1D). These results were consistent with the protein levels detected through western blotting and immunofluorescent staining (Fig. 1E–H).

To investigate if serial passages of cells have an impact on TLR expression, we analyzed TLR6 mRNA levels in GES-1 cells that were untreated and those that had undergone 1st, 10th, 20th, and 30th passages. Our results demonstrated that there was no significant difference in TLR6 mRNA expression across different stages of GES-1 cells. (Supplementary Fig. 1A).

All of the findings showed that the TLR6 expression is typically upregulated during the acute phase of *H. pylori* infection, but decreases over time during the chronic phase. Sustained exposure to *H. pylori* or *H. pylori* lysate was found to inhibit TLR6 expression in GES-1 cells.

### *H. pylori* and *H. pylori* lysate induced immune tolerance of GES-1 cells with decreasing inflammatory cytokine release following prolonged coculture

**Fig. 2:**
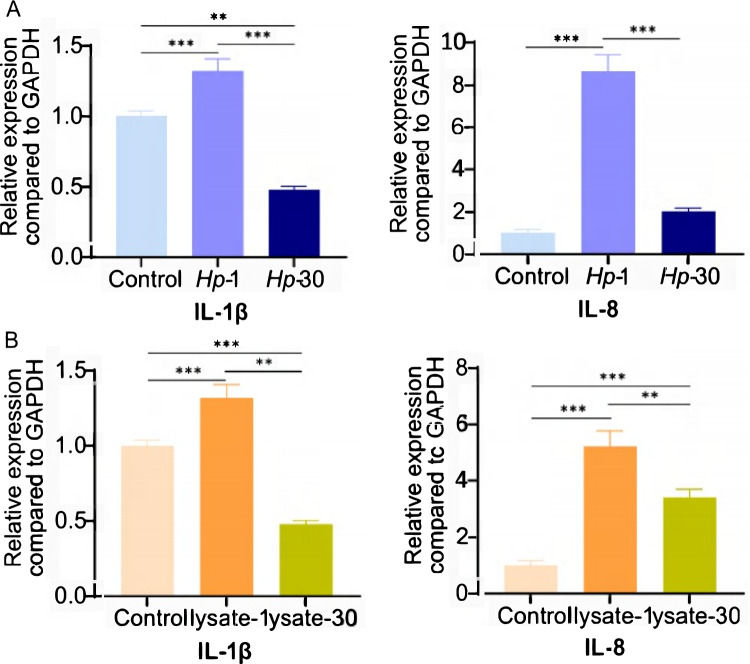
IL-1β mRNA expression (**A**) and IL-8 mRNA expression (**C**) in control cells, *Hp-*1 cells and *Hp-*30 cells. IL-1β mRNA expression (**B**) and IL-8 mRNA expression (**D**) in control cells, lysate-1 cells and lysate-30 cells. All experiment were performed in triplicate and all data represents means ± SEM. ***P < 0.001, **P < 0.01

TLRs play a crucial role in regulating the expression of inflammatory factors, which in turn helps to control the level of inflammation [39]. In order to investigate this further, we conducted a comparison of the levels of various *H. pylori* infection-related inflammatory cytokines using Luminex multiplex in GES-1 cells, lysate-1 cells, and lysate-30 cells. Our findings revealed that the levels of IL-8, IL-1β, Fas, IFN-γ, IL-23, CD163, and CD66a were significantly higher in lysate-1 cells when compared to GES-1 cells. However, these levels were observed to decrease in lysate-30 cells (Supplementary Fig. 1). We performed qPCR analysis of mRNA levels in wild-type cells, *Hp*-1 cells and *Hp*-30 cells, as well as in lysate-1 cells and lysate-30 cells, given that IL-1β has a direct positive relationship with the expression of TLR6 and IL-8 is one of the major cytokines secreted by epithelial cells inducing neutrophil chemotaxis [40, 41]. In comparison to wild-type cells and *Hp*-30 cells, the results showed that *Hp*-1 cells exhibited the highest quantities of IL-1β and IL-8 mRNA (Fig. 2A). When GES-1 cells were treated with *H. pylori* lysate, the same outcomes were seen (Fig. 2B). In untreated GES-1 cells and GES-1 cells from the 1st and 30th passages, the expression level of IL-1β and IL-8 remained unchanged, indicating that cell culture passage periods did not significantly affect the immune response (Supplementary Fig. 2B, C). The findings show that either *H. pylori* or *H. pylori* cell lysate resulted in the development of an inflammatory tolerance in GES-1 cells.

### *H. pylori* activates JNK inflammatory signaling pathway through TLR6

TLR6 forms heterodimers with TLR2 and is capable of recognizing various of microbial ligands [42]. Recent studies have shown that TLR6/NF-κB pathway is activated in *Mycoplasma gallisepticum* (*M. gallisepticum*)-infected chickens, whereas the TLR6/MAPK signaling pathway is involved in the activation of porcine alveolar macrophages [43–45]. We forced all TLR6 signaling pathways from the KEGG database (http://www.kegg.jp/) in order to better understand the function of TLR6 in *H. pylori*-induced immunological tolerance. TLR6 signaling mediates the proinflammatory response via two major downstream pathways, the PI3K/NF-κB and MAPK pathways.

**Fig. 3:**
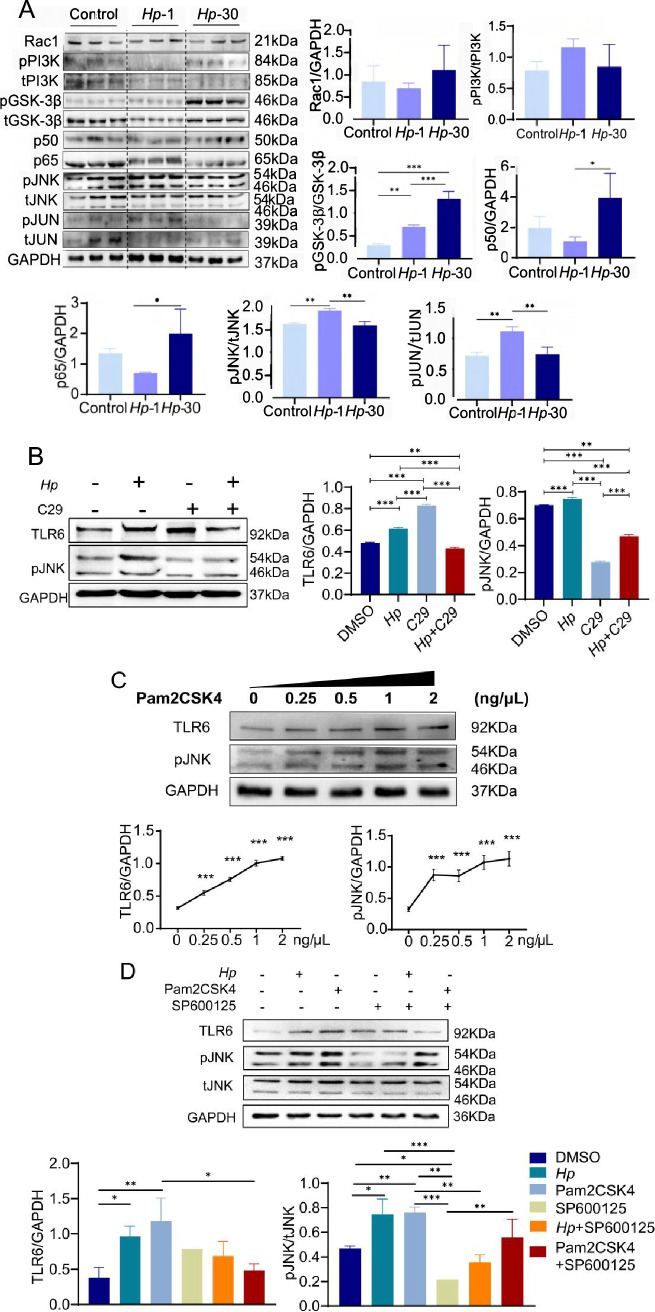
Rac1, pPI3K, tPI3K, pGSK-3β, tGSK-3β, NF-κB p50 and NF-κB p65, pJNK, tJNK, pJUN and tJUN expression in control cells, *Hp-*1 cells and *Hp-*30 cells (**A**). TLR6 and pJNK expression in control cells, *Hp-*1 cells, GES-1 cells treated with 1 μM C29 for 24 h and GES-1 cells treated with *H. pylori* and 1 μM C29 for 24 h (**B**). TLR6 and pJNK expression in control cells, GES-1 cells treated with 0.25 μg/mL, 0. 5 μg/mL, 1 μg/mL and 2 μg/mL Pam2CSK4 for 24 h (**C**). TLR6 and pJNK expression in GES-1 cells, *Hp-*1 cells, GES-1 cells treated with 1 μg/mL Pam2CSK4 for 24 h, GES-1 cells treated with 2 μg/mL SP600125 for 24 h, GES-1 cells treated with *H. pylori* and 2 μg/mL SP600125 for 24 h and GES-1 cells treated with 1 μg/mL Pam2CSK4 and 2 μg/mL SP600125 for 24 h (**D**). All experiment were performed in triplicate and all data represents means ± SEM. ***P < 0.001, **P < 0.01, *P < 0.05

The protein levels of important components in the TLR6 signaling pathway were subsequently examined by western blot. *Hp*-1 cells had higher levels of JNK and JUN phosphorylation than untreated GES-1 cells or *Hp*-30 cells, although Rac1, PI3K, GSK-3, NF-κB p50, and p65 levels remained unchanged (Fig. 3A). According to the phosphorylated JNK and JUN expression patterns, TLR6 is the mechanism via which *H. pylori* activates the JNK/JUN pathway rather than the Rac1/PI3K/NF-κB pathway or the PI3K/GSK-3β pathway.

To test the hypothesis, we used TLR2/6 heterodimer inhibitor C29, TLR2/6 heterodimer agonist Pam2CSK4, and JNK inhibitor SP600125. Our findings indicate that C29 effectively inhibited the activation of JNK in *H. pylori* infected GES-1 cells (Fig. 3B). Furthermore, when we treated GES-1 cells with varying concentrations of Pam2CSK4 and measured the activation of the JNK pathway using western blotting, we observed a dose-dependent response to Pam2CSK4 similar to TLR6 (Fig. 3C). We then treated GES-1 cells with Pam2CSK4 and SP600125. SP600125 showed significant inhibition of JNK phosphorylation, which was partially restored by *H. pylori* and Pam2CSK4 (Fig. 3D). All of the results confirmed that *H. pylori* activates JNK signaling pathway through TLR6.

To further investigate the correlation between TLR6 and inflammatory cytokines, we established a TLR6 knockout HEK293t cell line (KO) and exposed the cells to *H. pylori* (KO + *Hp*) and Pam2CSK4 (KO + Pam). The Western blotting results revealed that the activation of JNK was absent in TLR6 knockout cells upon exposure to either *H. pylori* or Pam2CSK4 (Supplementary Fig. 3A). Conversely, in wild-type HEK293t cells (WT) infected with *H. pylori* (WT + *Hp*) and Pam2CSK4 (WT + Pam), there was a significant upregulation in the mRNA expression of IL-8, as well as an increase in the supernatant IL-8 content (Supplementary Fig. 3B, C). Notably, TLR6 knockout cells did not exhibit such an elevation. These findings substantiate the notion that *H. pylori* induces the expression of specific inflammatory cytokines, including IL-8, through the TLR6/JNK pathway.

### TLR2/6 heterodimer agonist enhances the sensitivity of H. pylori tolerant GES-1 cells

**Fig. 4:**
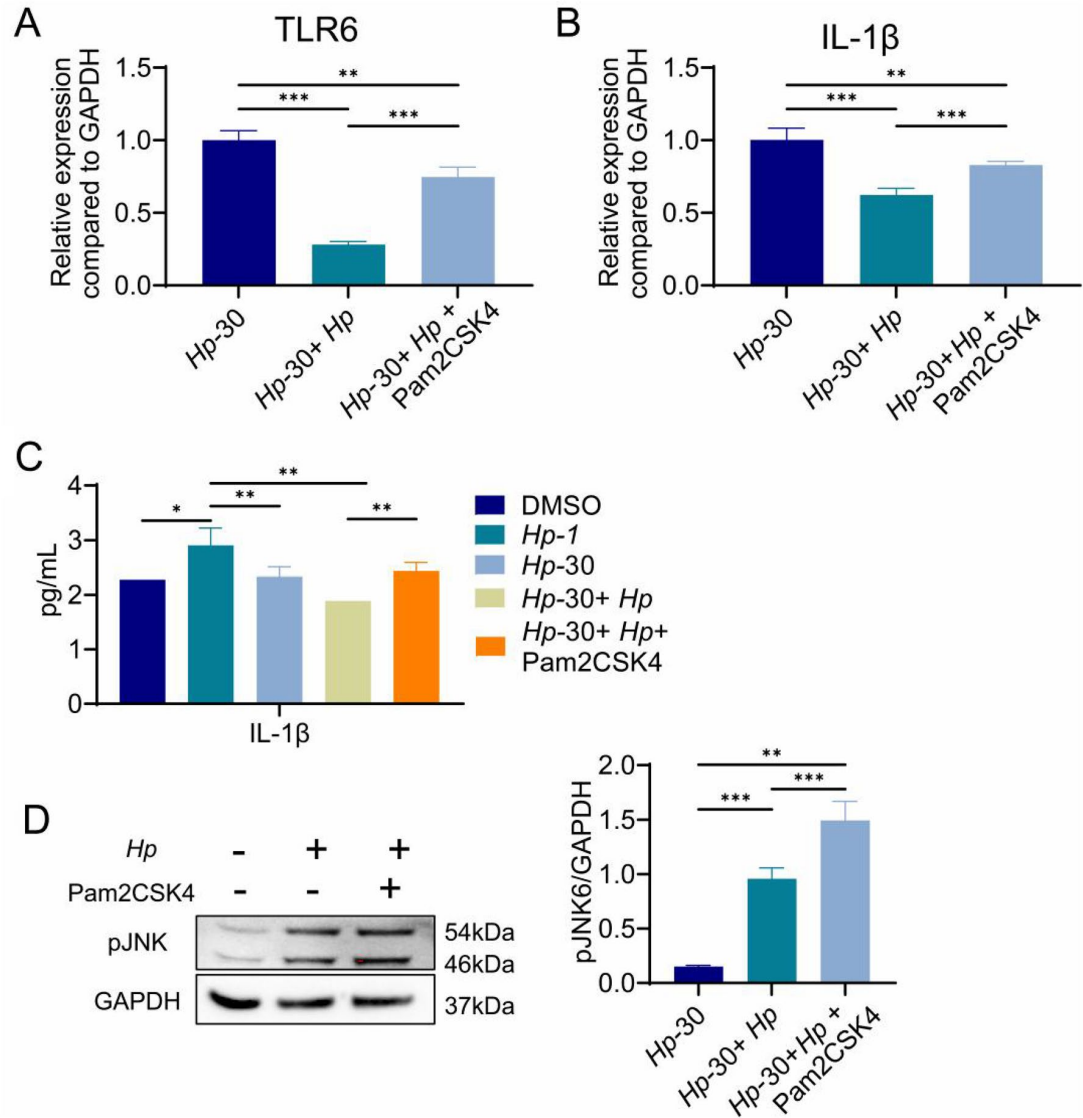
TLR6 mRNA expression (**A**) and IL-1β mRNA expression (**B**) in *Hp-*30 cells, *Hp-*30 cells co-culture with *H. pylori* for 24 h and *Hp*-30 cells co-culture with *H. pylori* and 10 μg/ml Pam2CSK4 for 24 h. Supernatant IL-1β content (**C**) in control cells, *Hp-*1 cells, *Hp*-30 cells, *Hp*-30 cells infected with *H. pylori* for 24 h and *Hp*-30 cells co-culture with *H. pylori* and 1 μg/ml Pam2CSK4 for 24 h. pJNK expression (**D**) in *Hp-*30 cells, *Hp-*30 cells co-culture with *H. pylori* for 24 h, *Hp*-30 cells co-culture with *H. pylori* and 1 μg/ml Pam2CSK4 for 24 h. All experiment were performed in triplicate and all data represents means ± SEM. ***P < 0.001, **P < 0.01, *P < 0.05

The aforementioned results demonstrated that sustained exposure to *H. pylori* or *H. pylori* lysate inhibit TLR6-mediated immune response. We wondered if treatment with Pam2CSK4 could restore TLR6 expression. As expected, Pam2CSK4 increase the mRNA level of TLR6 and IL-1β in *Hp-*30 cells in which *H. pylori* failed (Fig. 4A, B). Similar results were obtained when the levels of IL-1β were determined by ELISA in the supernatant of stimulated cells (Fig. 4C). In addition, the levels of phosphorylated JNK increased significantly in *Hp*-30 cells after Pam2CSK4 treatment, which confirmed that *H. pylori* regulates proinflammatory factor levels through the TLR6/JNK pathway (Fig. 4D). Overall, the data suggests that the TLR2/6 heterodimer agonist could restore the sensitivity of H. pylori tolerant GES-1 cells.

### TLR6 and gastric inflammation scores initially increased and then decreased in H. pylori-infected gerbils

**Fig. 5:**
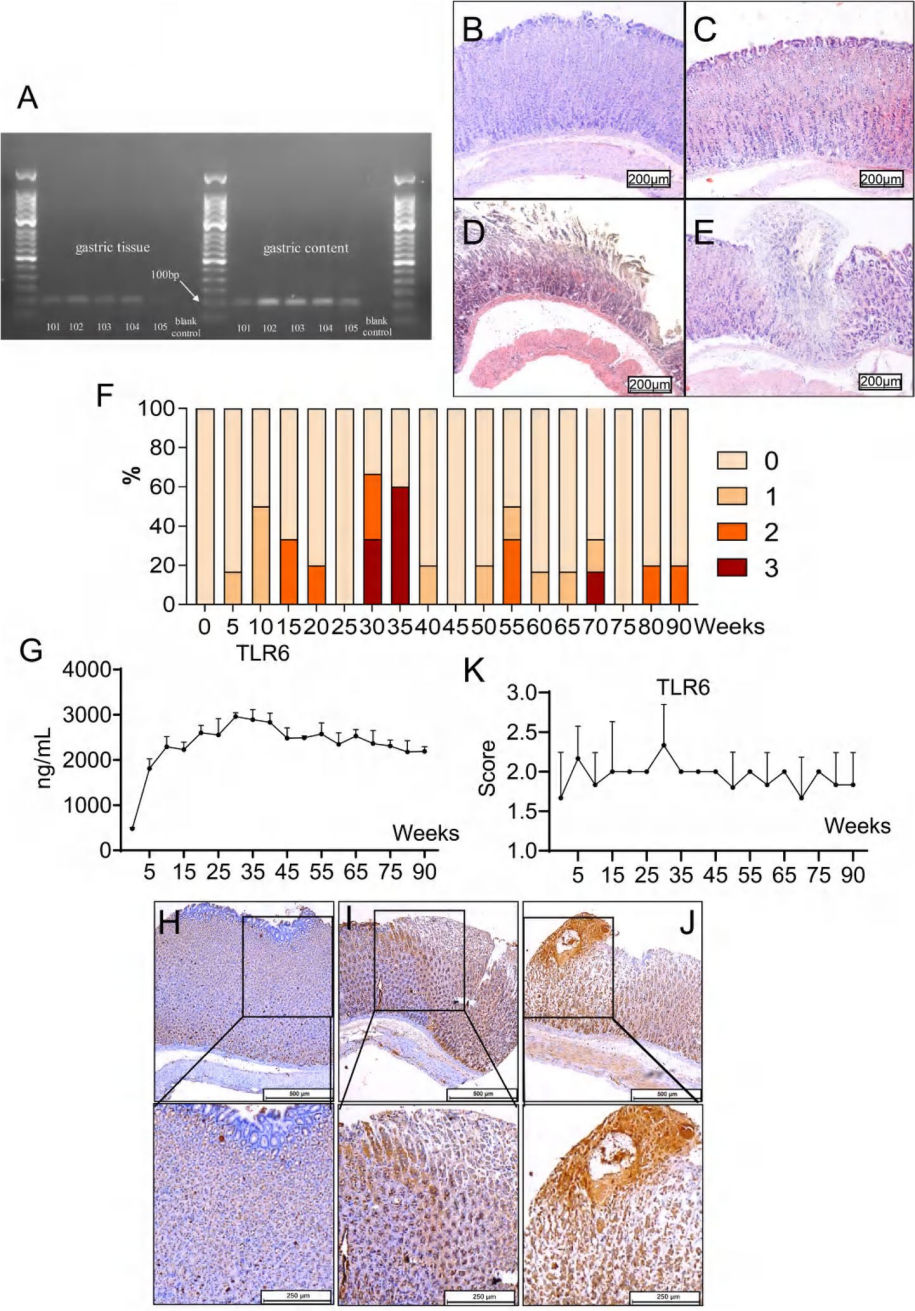

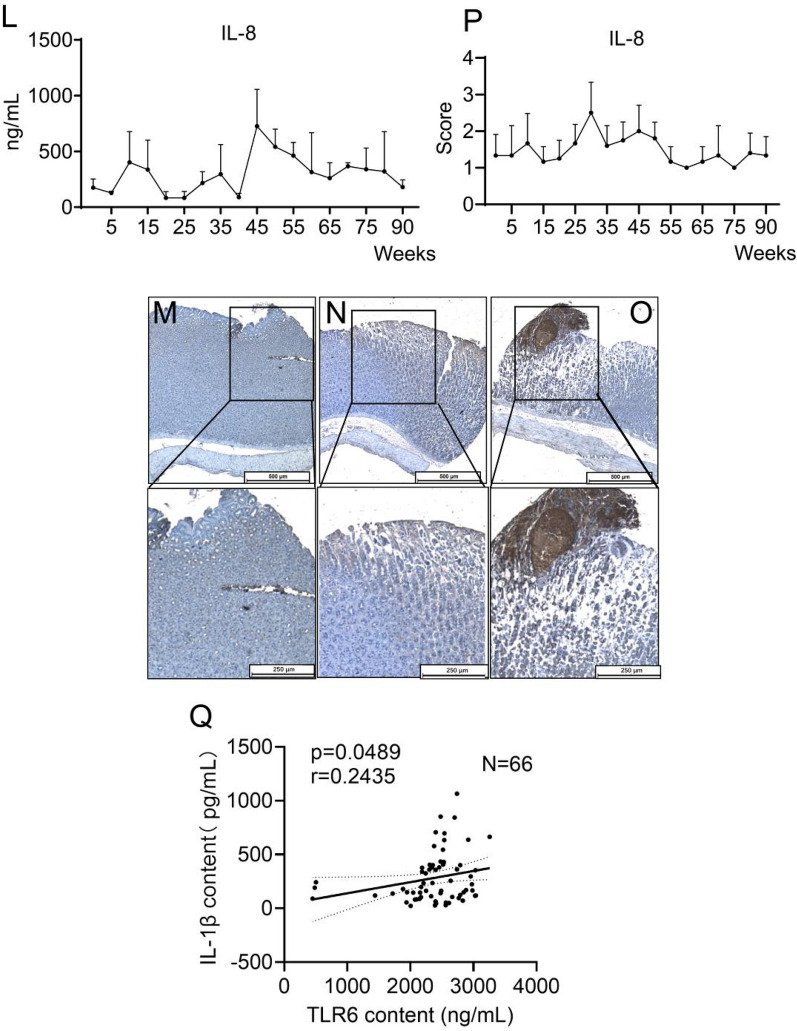
Electrophoresis (**A**) of nested PCR products for Mongolian gerbils’ gastric tissue and gastric content. The presence of a visible band at 100bp indicates a positive result for *H. pylori*-infection, while the absence of such a band indicates a negative infection. Nucleotide-free water was considered as the blank control. The number 1 ~ 5 represent to animal ID. The gastric mucosa of *H. pylori*-negative and *H. pylori*-positive Mongolian gerbils. H&E, × 100. Antrum biopsies from healthy non-infected gastric mucosa (**B**). No obvious lesion, Score = 0. Superficial gastritis (**C**), score = 1. Atrophic gastritis (**D**), score = 2. Dysplasia (**E**), score = 3. The percentage (**F**) of gerbils who developed gastric lesions after being infected with *H. pylori* from 0 to 90 weeks accounted for the total number of gerbils in each group. TLR6 expression (**G**) in the serum of *H. pylori* infected Mongolian gerbils. Each group was compared to the group of gerbils infected with *H. pylori* for 35 weeks. IHC staining of TLR6 expression (**H–J**) in *H. pylori* infected Mongolian gerbils’ gastric mucosa. Figure **H** is considered low positive (score = 1), Figure I is considered positive (score = 2), Figure **J** is considered high positive (score = 3). IHC score of TLR6 expression (**K**) in *H. pylori* infected Mongolian gerbils’ gastric mucosa. IL-8 expression (**L**) in the serum of *H. pylori* infected Mongolian gerbils. IHC staining of IL-8 expression (**M–O**) in *H. pylori* infected Mongolian gerbils’ gastric mucosa. Figure **M** is considered low positive (score = 1), Figure **N** is considered positive (score = 2), Figure **O** is considered high positive (score = 3). IHC score of IL-8 expression (**P**) in *H. pylori* infected Mongolian gerbils’ gastric mucosa. Correlation analyses (**Q**) of serum TLR6 and IL-1β levels in *H. pylori* infected Mongolian gerbils

To investigate the impact of continuous exposure to *H. pylori* on the gastric mucosa in vivo, we administered the bacterium to gerbils. We confirmed its presence in the gastric mucosa of the gerbils through isolation and culturing, followed by nested PCR testing of the gastric content and tissue [46]. The results showed visible target bands of the *H. pylori* specific urease gene at approximately 100 bp. Figure 5A demonstrates that all 96 gerbils were positive for *H. pylori*. The individual gerbils were graded based on their pathological scores, which ranged from 0 (no overt lesion) to 3 (dysplasia), with grades 1 and 2 representing superficial gastritis and atrophic gastritis, respectively (Fig. 5B–E). Out of the gerbils infected with *H. pylori* for 5 to 25 weeks, 24.14% (7/29) showed gastric lesions, with atrophic gastritis being the most severe lesion observed in this group (Supplementary Table 1). The prevalence of gastric lesions in gerbils infected with *H. pylori* for 30 to 35 weeks was 63.64% (7/11). However, out of the 56 gerbils infected with *H. pylori* for 40 to 90 weeks, only one developed dysplasia and the incidence of gastric lesions decreased to 19.64% (11/56) (Fig. 5F).

Our study found fluctuations in serum TLR6 levels during various infection durations and pathological lesions. TLR6 levels showed a significant increase during the first 35 weeks of infection in a time-dependent manner, followed by a gradual decrease (Fig. 5G). This trend was also observed in the IHC staining of TLR6 in gerbil gastric tissue (Fig. 5H–K). Similarly, IL-1β levels in the serum of *H. pylori*-infected gerbils and IHC staining of IL-8 on gerbil gastric mucosa showed a similar pattern (Fig. 5L-P). A positive correlation was found between serum IL-1β levels and serum TLR6 levels (n = 66, p = 0.0489) (Fig. 5Q), indicating that *H. pylori* could regulate the expression of TLR6 and affect the secretion of proinflammatory cytokines and immune response.

Taken together, our study suggests that in gerbils infected with *H. pylori*, the inflammatory response is initially severe in the acute stage but gradually decreases and remains at a low level if gastritis does not progress to intestinal metaplasia or gastric adenocarcinoma.

### TLR2/6 heterodimer agonist activated immune response and reduced the colonization of *H. pylori* in the gastric mucosa of gerbils

**Fig. 6:**
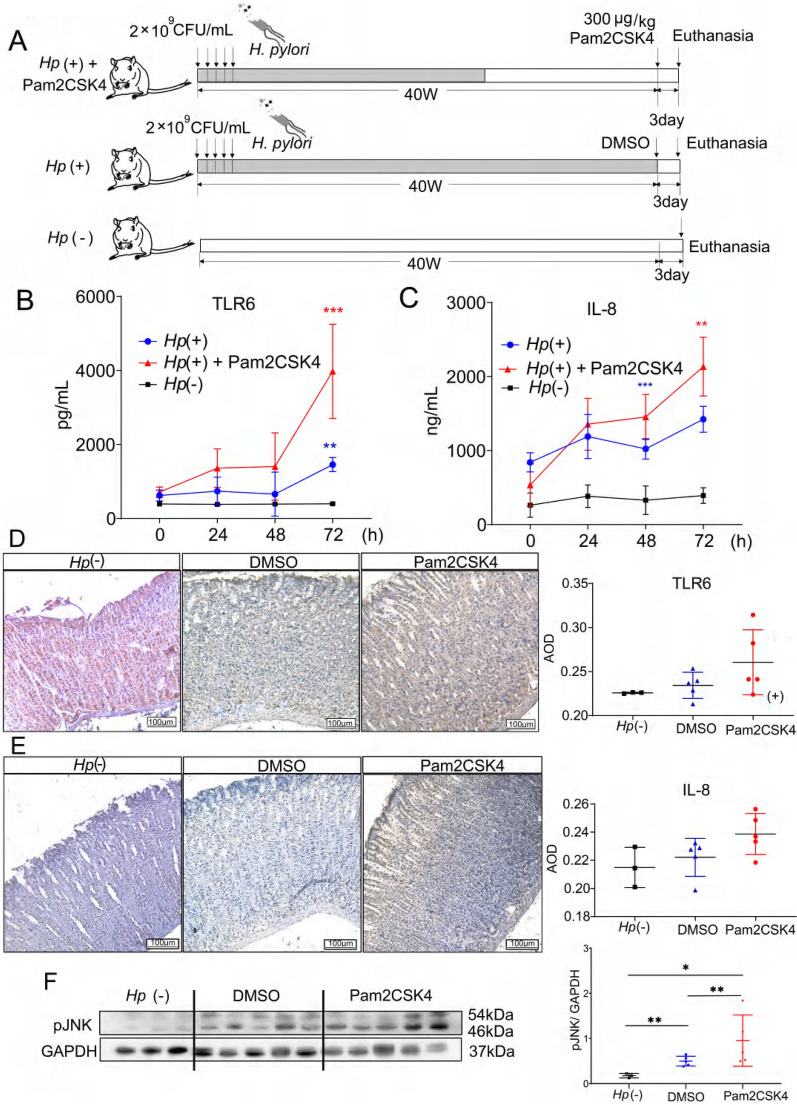

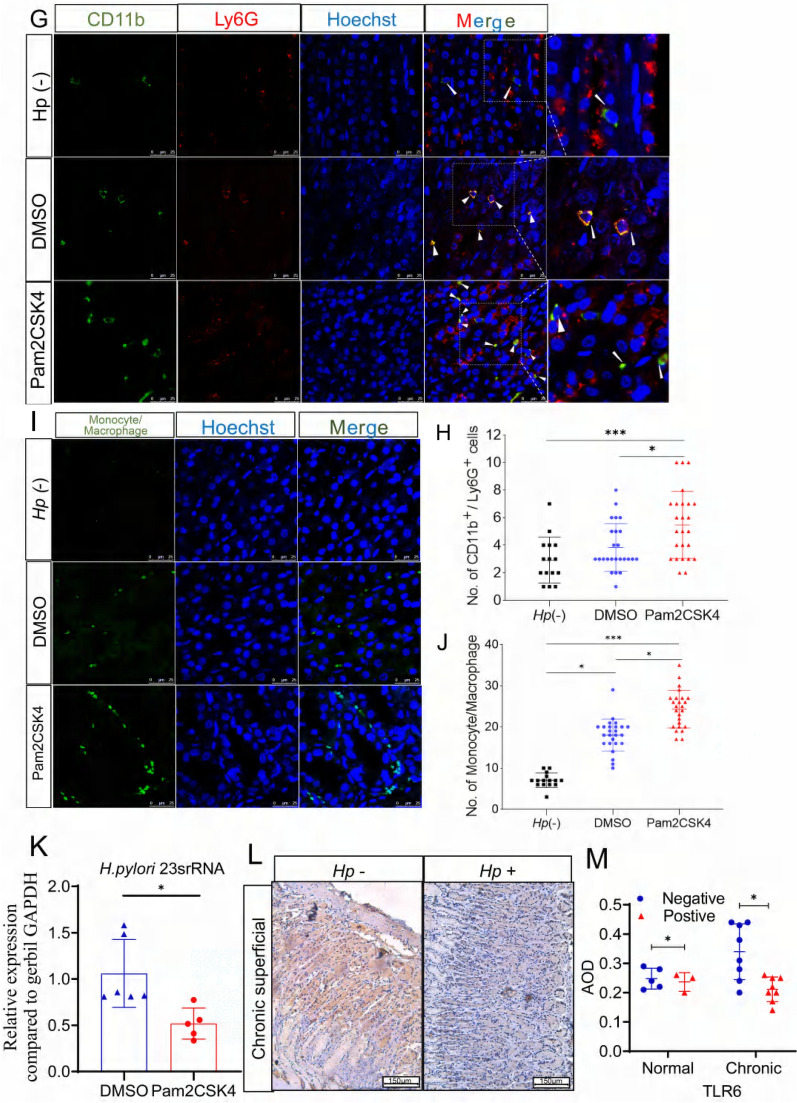
Schematic (**A**) of the in vivo treatment experimental plan. Serum TLR6 and IL-8 expression (**B, C**) and gastric mucosa TLR6 and IL-8 IHC staining (**D**, **E**) of the *H. pylori-*negative Mongolian gerbils or in the *H. pylori-*infected gerbils after 24 h, 48 h and 72 h intraperitoneal administration of Pam2CSK4 or DMSO. pJNK expression (**F**) in the gastric mucosa of *H. pylori-*negative Mongolian gerbils or in the *H. pylori-*infected gerbils after 72 h intraperitoneal administration of Pam2CSK4 or DMSO. The Immunofluorescence staining of CD11b^+^/Ly6G^+^ cells (**G**), monocyte/macrophage (**I**), the number of CD11b^+^/Ly6G^+^ cells (**H**) and the number of monocyte/macrophage (**J**) in gastric mucosa of *H. pylori*-negative gerbils, *H. pylori*-positive gerbils treated with DMSO or Pam2CSK4. The level of *H. pylori* 23srRNA (**K**) in gastric mucosa of *H. pylori*-positive Mongolian gerbils treated with DMSO or Pam2CSK4. IHC staining of TLR6 expression (**L**) in the gastric mucosa of patients. × 100. The average optical density (AOD) of TLR6 expression (**M**) in patients’ gastric mucosa. All experiment were performed in triplicate and all data represents means ± SEM. ***P < 0.001, **P < 0.01, *P < 0.05

In this study, we utilized a gerbil model infected with *H. pylori* to investigate the impact of a TLR2/6 heterodimer agonist on immune tolerance in vivo. After 40 weeks of infection, gerbils were administered with 300 μg/kg Pam2CSK4 or dimethylsulfoxide (DMSO) intraperitoneally (Fig. 6A). *H. pylori*-negative gerbils were used as the control group. Our results showed significantly higher levels of TLR6 and IL-8 in the serum of the Pam2CSK4 group 72 h after injection (Fig. 6B, C). IHC staining was performed to detect the expression of TLR6 and IL-8 in gerbils’ gastric mucosa. Although there was an increasing trend observed in the Pam2CSK4 group, there was no statistical significance (Fig. 6D, E). The activation of the TLR6/JNK signaling pathway was confirmed by the increased phosphorylation level of JNK in the Pam2CSK4 group (Fig. 6F).

In order to study the elimination of *H. pylori*, we analyzed the production of the neutrophil attractant IL-8 secreted by gastric epithelial cells. We employed IF staining to identify the presence of Ly6G and CD11b, which are neutrophil markers [47]. Pam2CSK4 injection promoted the infiltration of Ly6G^+^ and CD11b^+^ cells into the gastric mucosa in *H. pylori*-infected gerbils (Fig. 6G, 6H). The results of IF staining showed a positive correlation between Pam2CSK4 and increased monocyte/macrophage recruitment (Fig. 6I, J). Tuzun et al., reported amplification of *H. pylori* 23s rRNA in gerbil gastric mucosa, with relative fold changes calculated using gerbil GAPDH as an internal reference gene [48, 49]. In the study, it was observed that the increase in TLR6 expression using an agonist led to the inhibition of the colonization of *H. pylori* in gerbils (Fig. 6K). The findings indicate that TLR2/6 heterodimer agonists activate host immune responses through TLR6/JNK pathways, which can aid in the clearance of *H. pylori* infection in cases of continuous exposure.

### *H. pylori* infection suppressed the expression of TLR6 in the gastric mucosa of patients with gastritis

To examine the expression levels of TLR6 in the gastric mucosa of clinical patients, we conducted a validation study using a sample size of 24 gastric mucosa tissue samples from Beijing Shijitan Hospital. The samples were divided into four groups based on their *H. pylori* infection and pathological status: *Hp*^+^ normal group (n = 3), *Hp*^*−*^ normal group (n = 5), *Hp*^+^ chronic superficial gastritis group (n = 8) and *Hp*^*−*^ chronic superficial gastritis group (n = 8). In patients with chronic superficial gastritis, IHC staining demonstrated decreased TLR6 staining in *H. pylori*^+^ gastric mucosa compared with *H. pylori*^*−*^ gastric mucosa, which confirms that the colonization of *H. pylori* suppressed the expression of TLR6 in the gastric mucosa of patients with gastritis (Fig. 6L, M).

## Discussion

*H. pylori* can establish persistent colonization by inhibiting the host immune response to escape from the host immunity [50]. Researchers have found that the immune escape of *H. pylori* can occur in different ways [51, 52]. TLRs play a critical role in the maintenance of immune tolerance to commensal bacteria by detecting pathogen-associated molecular patterns (PAMPs) [26, 53]. In our study, we found that long-term exposure to *H. pylori* downregulated TLR6 expression and reduced the levels of downstream inflammatory cytokines in GES-1 cells. This is also observed in *H. pylori*-positive gerbils, which have similar tumor progression associated with *H. pylori* to that of humans [54, 55]. Clinical gastric mucosa tissue specimen analysis confirmed that low levels of inflammation are accompanied by downregulated TLR6 expression in the late stages of *H. pylori* infection. It helped *H. pylori* escape eradication, which was beneficial to the colonization and proliferation of bacteria and therefore contributed to the development of gastric mucosal lesions. Taken together, we are convinced that long-term stimulation of *H. pylori* changes the sensitivity of GES-1 cells to pathogen components following *H. pylori* infection through TLR6 signaling.

TLR6 forms heterodimers with TLR2 to recognize different configurations of lipoproteins [42]. The TLR6/MyD88/NF-κB pathway is activated in *M. gallisepticum*-infected chickens, while the TLR6/NOX2/MAPK signaling pathway is involved in the activation of porcine alveolar macrophages [43–45]. The effects of long-term *H. pylori* infection or *H. pylori* lysate stimulation on gastric epithelial cells have not yet been studied. In the current study, multiplex assays and real-time PCR results showed that the expression of inflammatory cytokines, such as IL-1β and IL-8, was positively correlated with TLR6 levels. Real-time PCR and western blotting results suggested that sustained exposure to *H. pylori* reduced the levels of JNK and JUN phosphorylation that were increased after short-term infection with *H. pylori.* All the data showed that *H. pylori* colonized in the gastric mucosa regulates the inflammatory response through TLR6/JNK signaling pathway induced by the bacteria.

To elucidate the TLR6 signaling pathway, we introduced several small molecule inhibitors and agonists. TLR2/6 heterodimers inhibitor C29 suppressing the expression of pJNK in *H. pylori* infected GES-1 cells, which confirms that *H. pylori* activates the JNK signaling pathway. Pam2CSK4 is known to activate TLR2/6 heterodimers and increase the expression of TLR6, ultimately increasing the expression of downstream inflammatory cytokines [56]. Therefore, we treated GES-1 cells with the Pam2CSK4 to mimic *H. pylori* combined with JNK inhibitor SP600125 to verify the role of the TLR6 signaling pathway in GES-1 cells. It has been strongly established that *H. pylori* affects the expression of inflammatory cytokines in GES-1 cells through the TLR6/JNK pathway. This conclusion was further confirmed in TLR6 knockout HEK293T cells, as Western blotting indicated that the JNK pathway was not activated in these cells by *H. pylori* or Pam2CSK4. In wild-type cells, increased IL-8 expression level was observed but not in TLR6 knockout cells.

During the proliferation and metabolism process of bacteria colonizing the stomach, a large number of lytic or disintegration products are produced and released [57]. Studies have reported that soluble peptidoglycan induces NF-κB-dependent reporter gene activation in TLR2-expressing cells, and the effect is more pronounced at lower soluble peptidoglycan (sPGN) concentrations [58]. When we treated GES-1 cells with different concentrations of Pam2CSK4 and found that pJNK exhibited a dose-dependent response to Pam2CSK4. However, the specific components of *H. pylori* that activate the expression of TLR6 are still unknown. The TLR6 signaling pathway and the specific mechanism involved in decreased sensitivity to *H. pylori* should be further elucidated when we fully identified the specific substances in *H. pylori*.

Targeting TLRs has been proposed as a novel alternative therapeutic approach in the diagnosis and therapy of different conditions, such as rheumatoid arthritis, multiple sclerosis, psoriasis, and colitis [59, 60]. Hence, we proposed that TLR6 agonists may activate the inflammatory pathway and subsequently promote the elimination of pathogens by the host immune system during the later stages of infection. TLR6 agonists have been considered potential therapeutic drugs for thromboinflammatory diseases [55]. The inhaled drug PUL-042, which is composed of synthetic ligands for TLR2/6 (Pam2CSK4) and TLR9 (ODN M362), is in the clinical stage [61]. In this research, we found that Pam2CSK4 has the ability to restore the expression of TLR6 in GES-1 cells. In addition, Pam2CSK4 reduced the colonization of *H. pylori* in the gastric mucosa of gerbils through the activation of the TLR6 signaling pathway that leads to increased secretion of IL-8 in epithelial cells and the recruitment of neutrophils. TLR6 agonists can be considered immunotherapy agents in *H. pylori* infection.

**Fig. 7:**
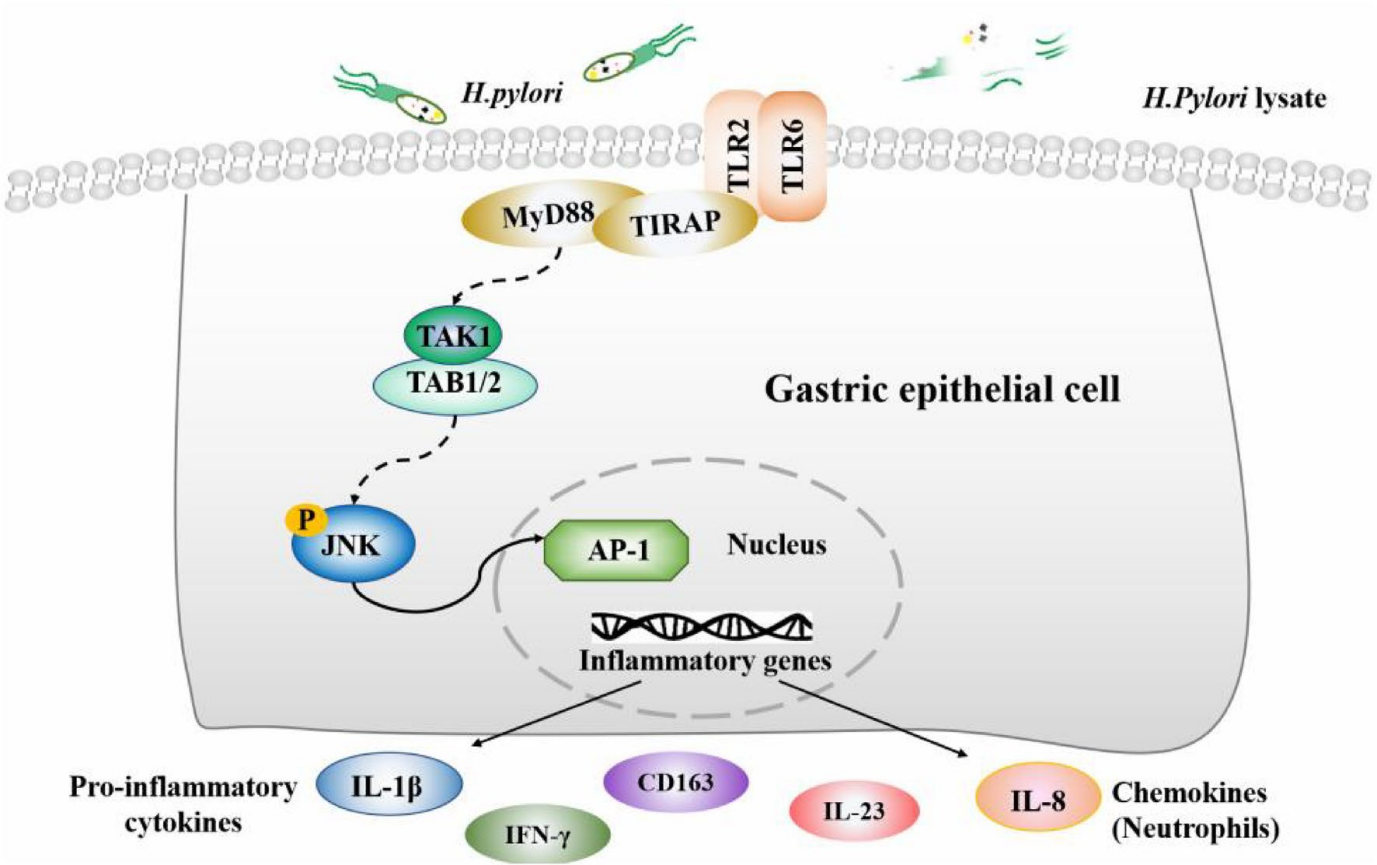
A Model of the TLR6 signal pathway

In conclusion, we utilized a co-culture model of GES-1 cells and *H. pylori* or *H. pylori* lysate to simulate *H. pylori* infection in vivo. Our findings indicate a significant increase in TLR6 expression and inflammatory cytokine production during the early stages of co-culture, followed by a gradual decline after the initial peak. These results were further validated in both *H. pylori*-infected gerbils and clinical patients with *H. pylori* infection and gastritis. The immune escape mechanism of *H. pylori* is achieved by inhibiting the TLR6/JNK signaling pathways that are activated by *H. pylori* (Fig. 7). It has been observed that TLR6 agonists can decrease bacterial colonization in gerbils. We suggest that TLR6 could be a promising target for immunotherapy to eradicate *H. pylori* infections.

## Materials and methods

### Cell culture

The human gastric epithelial cell line GES‐1 was purchased from the Beijing Dingguo Changsheng Biotechnology Co., Ltd. (Beijing, China). Cells were cultured in DMEM (Gibco, NY, USA) supplemented with 10% fetal bovine serum (Gibco, NY, USA) and 1% penicillin streptomycin solution (HyClone, UT, USA) at 37 °C under 5% CO_2_ conditions.

### Bacterial culture and preparation of *H. pylori* lysate

The *H. pylori* strain ATCC43504 (CagA^+^/VacA^+^), provided by the National Institutes for Food and Drug Control, was cultured on Columbia blood agar base medium (Oxoid, Hampshire, England)-coated plates supplemented with 5% defibrinated sheep blood (Fujian Sanqiang Biochemical Co., Ltd., Fujian, China) at 36 °C for 48 h under microaerophilic conditions (10% CO_2_). The *H. pylori* culture was harvested and then suspended in phosphate-buffered saline (PBS) solution (Solarbio, Beijing, China) and adjusted to a concentration of 6 × 10^6^ CFU/mL.

The *H. pylori* suspension was placed on ice and sonicated for 30 s at 100 W power for a total of 10 times with intervals of 20 s each. Suspensions were centrifuged at 12,000×*g* for 10 min. The supernatant was removed, and the protein concentration was measured using a BCA protein quantitation kit (Thermo, MA, USA). The supernatant was diluted to 200 μg/mL with PBS and was considered *H. pylori* lysate.

### Cell culture with *H. pylori* or *H. pylori* lysate

GES-1 is a non-malignant gastric cell line which was originated from health human gastric mucosa, which is commonly used to study the pathogenic mechanism of *H. pylori* [62]. The cell line was purchased from Beijing Dingguo Changsheng Biotechnology Co., Ltd.

GES-1 cells were counted with a hemocytometer, and then 6 × 10^5^ cells were seeded in 10 cm dishes. To ensure that the individual GES-1 cells are fully infected, *H. pylori* strain ATCC43504 (6 × 10^8^ CFU) were added to GES-1 cells at a ratio of 1000:1 between bacteria and cells. After a 24 h of incubation in an aerobic environment (5% CO_2_, 21% O_2_, 74% N_2_), the medium was discarded, and then the cells were washed five times with 3 mL phosphate-buffered saline (PBS) to remove free bacteria as much as possible. Subsequently, cells recovered in *H. pylori*-free fresh DMEM medium for 24 h. The process was repeated 30 times until GES-1 cells cocultured with *H. pylori* for 30 generations (*Hp*-30) were obtained.

Method of GES-1 cells and gastric cancer cell line MKN-45 co-cultured with *H. pylori* lysate was previously described [34]. In brief, 6 × 10^5^ cells were plated in a 10 cm dish. DMEM medium with 7 μL *H. pylori* lysate (2 mg/mL) was added to the plates for a total of 7 mL. The culture medium was discarded 24 h after the incubation of cells. The cells were washed for three times with PBS and digested with 0.5% trypsin. Then 6 × 10^5^ cells were taken and re-seeded in a new 10 cm dish, and fresh DMEM medium with *H. pylori* lysate was added again. Repeated this process 30 times to obtain lysate-30 cells and MKN-45 + lysate F30 cells.

The HEK293T cell line and the TLR6 knockout HEK293T cell line were purchased from Cyagen Biotechnology Co., Ltd. The *H. pylori* strain ATCC43504 (6 × 10^8^ CFU) was added to the cells at a ratio of 100:1 between bacteria and cells.

### Multiplex assay

The supernatants of untreated GES-1 cells (GES-1), GES-1 cells exposed to *H. pylori* lysate for 1 generation (lysate-1) and 30 generations (lysate-30) cells were collected and used for cytokine determination in Luminex assays. The levels of TNF-α, CCL-20, IL-8, CCL-28, CXCL-2, IFN-γ, SLPI, IL-23, GDF-15, IL-11, CD-163, IL-1β, EGF, HB-EGF, VEGF-α, CD66α, FAS, TFPI, and IGFBP-1 were measured using technical microspheres with a Luminex X-200 instrument (Luminex, TX, USA).

### RNA extraction and real-time PCR analysis

Total mRNA was extracted from GES-1 cells and gastric tissue using RNA isolater Total RNA Extraction Reagent (Vazyme, Nanjing, China, R401-01) and subjected to reverse transcription using 5 × All-In-One RT MasterMix (ABM, Richmond, BC, Canada). Real-time PCR (qPCR) was performed on a CFX96 Real-Time PCR detection system (Bio–Rad Laboratories, Inc., Hercules, CA, USA; cat. no. 185-5195) using AceQ qPCR SYBR Green Master Mix or Taqprobe qPCR Master Mix (ABM, Richmond, BC, Canada). All reactions were performed in triplicate. Relative changes in transcript levels were normalized to GAPDH mRNA and were calculated using the ^ΔΔ^Ct method. Sequence-specific primers are shown in Supplementary Table 2.

### Western blotting analysis

Total proteins of GES-1 cells were extracted by RIPA Lysis Buffer (Solarbio, Beijing, China), separated by 10% sodium dodecyl sulfate–polyacrylamide gel electrophoresis, and then transferred onto nitrocellulose membranes (Millipore, MA, USA). Blots were blocked in 10% dry milk-TBST (20 mM Tris–HCl [pH of 7.6], 127 mM NaCl, 0.1% Tween 20) for 1 h at room temperature and incubated with the specific primary antibody at 4 °C overnight, followed by incubation with the secondary antibody (Solarbio, SE134) at room temperature for 1 h. The anti-TLR6 antibody (Proteintech, 22240-1-AP), anti-Rac1 antibody (Proteintech, 24072-1-AP), anti-phosphorylated PI3 kinase antibody (Abcam, ab182651), anti-NF-κB p105/p50 antibody (Abcam, ab32360), anti-NF-κB p65 antibody (Abcam, ab32536), anti-phosphorylated TAK1 antibody (Cell Signaling Technology, 9339S), anti-phosphorylated SAPK/JNK antibody (Cell Signaling Technology, 4668 T), anti-phosphorylated JUN antibody (Proteintech, 248,891–1-AP), and anti-GAPDH antibody (Cell Signaling Technology, 5174) were purchased.

The TLR2/6 heterodimer inhibitor C29 was purchased from MedChemExpress (Monmouth Junction, NJ, USA)(HY-100461). The TLR2/6 heterodimer agonist Pam2CSK4 was purchased from Apexbio (B5665). The JNK inhibitor SP600125 was purchased from MedChemExpress (HY-12041). For the in vitro experiments, the compounds were diluted to different working concentrations with DMEM medium.

### Animal experiments

A total of 107 conventional Mongolian gerbils (6–8 weeks old, weighing 50–60 g) were used to replicate the *H. pylori* infection model, and 9 *H. pylori*-free gerbils were used as the control group. All rodents were obtained from SPF (Beijing) Biotechnology Co., Ltd. and housed at a Level II biosafety laboratory in the Chinese Center for Disease Control and Prevention. All gerbils were maintained in cages on a 12-h light/dark cycle. The room temperature was maintained at 22–24 °C, and humidity was maintained at 60–70%. The animal experiments were conducted in accordance with the Guidelines of the CMU Animal Experiments and Experimental Animals Management Committee under a protocol approved by the Animal Experiments and Experimental Animal Welfare Committee of CMU (Permit number: AEEI-2016-154).

Gerbils were challenged with a single 0.5 mL dose containing 2 × 10^9^ CFU/mL *H. pylori* ATCC 43504 strain by gavage. The gerbils were fasted for 12 h prior to the challenge, and then oral gavage was performed 5 times at intervals of 48 h. Next, 5–6 gerbils were sacrificed after being anesthetized with CO_2_. The gastric tissue and blood were collected after the gerbils were euthanized.

### Gastric mucosa tissue specimens

Gastric mucosa samples were acquired from patients who received partial gastrectomy at the Surgery Centre of Diabetes Mellitus, Beijing Shijitan Hospital. None of the patients received radiotherapy or chemotherapy before surgery. The procedures related to human subjects were approved by the Ethics Committee of Beijing Shijitan Hospital. The pathological diagnosis was determined by an experienced pathologist. *H. pylori* infection was defined by a positive ^13^C urea breath test.

Patient samples were classified into the following six groups based on clinical histopathology information: *H. pylori*-positive normal group (n = 3), *H. pylori*-negative normal group (n = 5), *H. pylori*-positive chronic superficial gastritis group (n = 8), *H. pylori*-negative chronic superficial gastritis group (n = 8), *H. pylori*-positive peptic ulcer group (n = 11) and *H. pylori*-negative peptic ulcer group (n = 6). All tissues were paraffin‐embedded for subsequent experiments.

### DNA extraction, PCR, and electrophoresis

The DNA from the gerbils’ gastric tissue was extracted using a microsample genomic DNA extraction kit (Tiangen, Beijing, China). The nested PCR amplification primers were designed using the primer design software Primer Premier 5. The primers for the first PCR were F1: 5′-AGTAGGGCCATACATAGAAA-3′ and R1: 5′-GACAAAACTCGTAACCGT-3′. The expected PCR product was 499 bp. The primers for the second PCR were F2: 5′-CATAGTTGTCATCGCTTTT-3′ and R2: 5′-GCGTTGGTTGATAGGC-3′. The expected size of the PCR product was 100 bp.

The nested PCR amplification product was subjected to agarose gel electrophoresis (Amresco, OH, USA). For further details of the experimental setup, refer to the literature of Zhang et al. [46].

### Enzyme-linked immunosorbent assay (ELISA)

Blood of the *H. pylori-*infected gerbil was obtained from the orbital venous plexus, and then the serum was extracted by centrifugation. The cell culture supernatant was collected for ELISA. Serum and cell culture supernatant samples were subjected to ELISA detection following the ELISA kit manufacturer’s instructions. A gerbil TLR 6 ELISA kit was purchased from the Jiangsu Meibiao Biological Technology Company Limited (Jiangsu, China). A gerbil IL-1β ELISA kit, human Toll-like receptor 6 ELISA kit and human interleukin-1β ELISA kit were purchased from the Quanzhou Ruixin Biological Technology Co., Ltd. (Quanzhou, China).

### Histopathology

Gastric tissue of the Mongolian gerbils was fixed in 4% polyformaldehyde (PFA) for 1 day and transferred to 70% ethanol. Individual pyloric stomach biopsy material was placed in processing cassettes, dehydrated through a serial alcohol gradient, embedded in paraffin, and then cut into 4-μm-thick sections. Pyloric stomach sections were stained with hematoxylin and eosin (H&E). Two pathologists in a double-blind condition determined the pathologic status.

### Tissue microarray

A gastric tissue microarray, with 98 pyloric stomach tissue samples obtained from 95 *H. pylori*-infected gerbils and 3 *H. pylori*-free gerbils, was prepared by the Beijing Raisedragon Technology Development Co., Ltd.

### Immunofluorescence (IF) and immunohistochemistry (IHC) staining

The cell were immunofluorescently stained as follows: GES-1 cells were seeded in a confocal laser dish at 5 × 10^5^ cells/mL. After three washes in PBS, the cell were fixed in 4% paraformaldehyde for 10 min at room temperature and permeabilized for 30 min in PBS containing 0.2% Triton X-100 and 10% fetal bovine serum. Cells were incubated in primary antibodies overnight at 4 °C and then incubated with secondary antibodies for 2 h at room temperature. The nuclei were then counterstained with Hoechst dye and blocked with antifading mounting medium (Solarbio, Beijing, China). For paraffin sections, after dewaxing and dehydration, the following IF staining procedures were the same as the cell IF staining described above. IF staining was observed by laser scanning confocal microscopy (Lycra, Germany).

IHC staining was performed on the gastric paraffin sections of gerbils and patients. Paraffin sections were incubated overnight with primary antibodies at 4 °C, followed by incubation with biotinylated secondary antibodies (Zhongshan, Beijing, China) for 15 min at room temperature. Peroxidase activity was detected using 3,3′-diaminobenzidine (Zhongshan, Beijing, China). The sections were counterstained with hematoxylin, dehydrated, cover slipped with histomount reagent, and imaged using a microscope (Lycra, Germany).

The antibodies used for IF or IHC experiments were as follows: anti-TLR6 antibody (Abcam, ab37072), anti-CXCL8/IL-8 antibody (Proteintech, 27095-1-AP), anti-CD11b antibody (Proteintech, 21851-1-AP), neutrophil marker antibody (Santa Cruz, sc-59338), goat anti-rat IgG Alexa Fluor 594 antibody (Abcam, ab150160), monocyte/macrophage marker antibody (Santa Cruz, sc-52603) and goat anti-rabbit IgG Alexa Fluor 488 antibody (Huabio, HA1104).

### Statistical analysis

All statistical analyses were performed using GraphPad Prism 8 or Microsoft Excel. Data are presented as the mean ± SEM. Independent sample *t* test, one-way ANOVA, and multi-way ANOVA were used. Statistical significance was assumed if the *P* value was < 0.05.

### Supplementary Information

Below is the link to the electronic supplementary material.

Supplementary file1 (DOCX 3148 KB)
