# Supplementary material for: Sustained exposure to Helicobacter pylori induces immune tolerance by desensitizing TLR6
Source: Gastric Cancer. 2024 Feb 4;27(2):324–42. doi: 10.1007/s10120-023-01461-7 (PMC10896808; doi:10.1007/s10120-023-01461-7)
Supplement: Supplementary file 1 — Supplementary file1 (DOCX 3148 KB) [file 10120_2023_1461_MOESM1_ESM.docx]

**Supplementary Figure 1:**

**
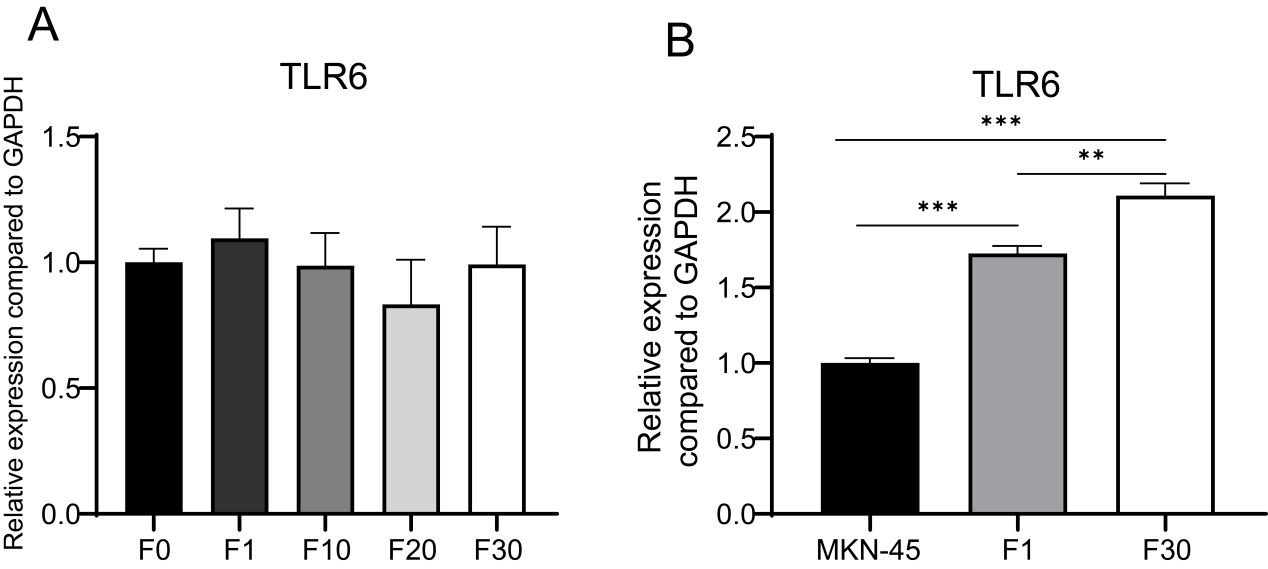
**

**Supplementary Figure 1:** TLR6 mRNA expression **(A)** in untreated GES-1 cells (F0), GES-1 cells at 1^st^ passage (F1), GES-1 cells at 10^th^ passage (F10), GES-1 cells at 20^th^ passage (F20) and GES-1 cells at 30^th^ passage (F30). TLR6 mRNA expression **(B)** in MKN-45 cells, MKN-45 cells+lysate F1 cells (F1) and MKN-45+lysate F30 cells (F30). All data represents means ± SEM. ***P <0 .001, **P <0 .01.

**Supplementary Figure 2:**

**Supplementary Figure 2:** The levels of IL-8, IL-1β, Fas, IFN-γ, IL-23, CD163 **(A)** in untreated GES-1 cells (control), lysate-1 cells and lysate-30 cells. IL-1β mRNA expression **(B)** and IL-8 mRNA expression **(C)** in untreated GES-1 cells (F0), GES-1 cells at 1^st^ passage (F1) and GES-1 cells at 30^th^ passage (F30).

**Supplementary Figure** 3**:**


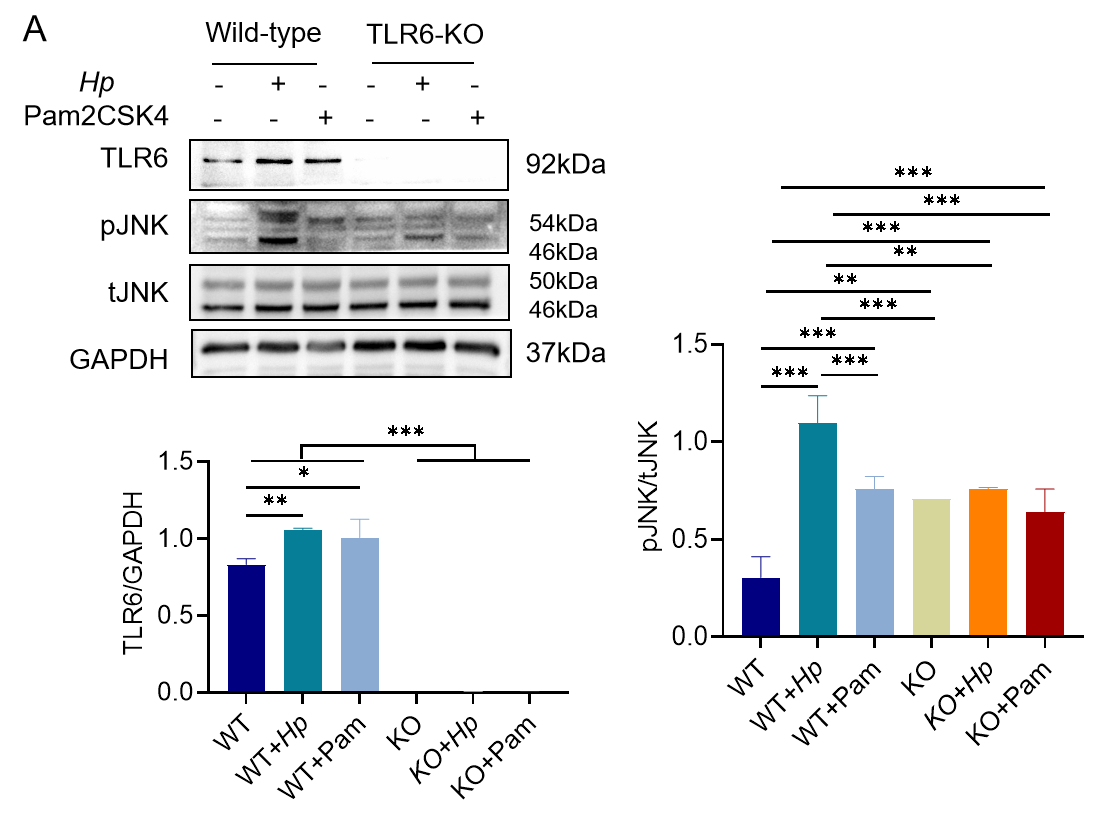


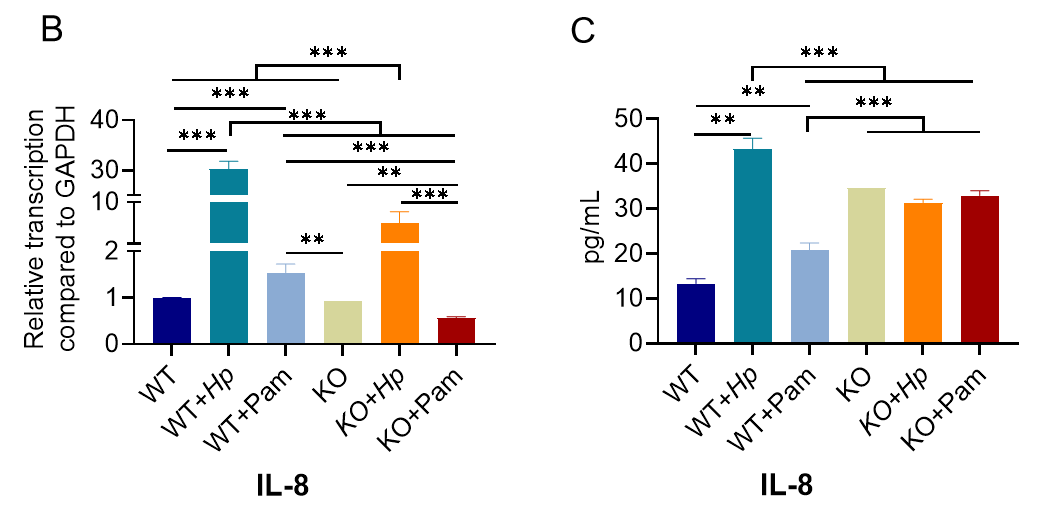


**Supplementary Figure 3**: pJNK and TLR6 expression (**A**) in WT cells, WT cells co-culture with *H. pylori* for 24 h (WT+*Hp*), WT cells co-culture with 2μg/ml Pam2CSK4 for 24 h (WT+Pam), KO cells, KO cells co-culture with *H. pylori* for 24 h (KO+*Hp*), KO cells co-culture with 2μg/ml Pam2CSK4 for 24 h (KO+Pam). IL-8 mRNA expression (**B**) and supernatant IL-8 content (**C**) in WT cells, WT+Hp cells, WT+Pam cells, KO cells, KO+***Hp*** cells, and KO+Pam cells. All experiment were performed in triplicate and all data represents means ± SEM. ***P < 0.001, **P < 0.01, * P < 0.05.

| Supplementary Table 1 | | | | | |
| --- | --- | --- | --- | --- | --- |
| Infection weeks | Dysplasia | Atrophic gastritis | Superficial gastritis | No typical lesion | Total number |
| 0 | 0 | 0 | 0 | 3 | 3 |
| 5 | 0 | 0 | 1 | 5 | 6 |
| 10 | 0 | 0 | 3 | 3 | 6 |
| 15 | 0 | 2 | 0 | 4 | 6 |
| 20 | 0 | 1 | 0 | 4 | 5 |
| 25 | 0 | 0 | 0 | 6 | 6 |
| 30 | 2 | 2 | 0 | 2 | 6 |
| 35 | 3 | 0 | 0 | 2 | 5 |
| 40 | 0 | 0 | 1 | 4 | 5 |
| 45 | 0 | 0 | 0 | 5 | 5 |
| 50 | 0 | 0 | 1 | 4 | 5 |
| 55 | 0 | 2 | 1 | 3 | 6 |
| 60 | 0 | 0 | 1 | 5 | 6 |
| 65 | 0 | 0 | 1 | 5 | 6 |
| 70 | 1 | 0 | 1 | 4 | 6 |
| 75 | 0 | 0 | 0 | 6 | 6 |
| 80 | 0 | 1 | 0 | 4 | 5 |
| 90 | 0 | 1 | 0 | 5 | 6 |
| Total number | 6 | 9 | 10 | 74 | 99 |

| Supplementary Table 2 The specific primers of TLR and GAPDH | | |
| --- | --- | --- |
|  | Sense primer ( 5'－3') | Antisense primer ( 5'－3') |
| TLR1 | ATTCAGTTTCCCACCCATCG | GCCAGCCCTCTAACACTTCA |
| TLR3 | TCCACCACCAGCAATACAAC | AAGCCAAGCAAAGGAATCG |
| TLR4 | CTCCTGCGTGAGACCAGAAA | CCGTGATAAAACGGCAGCAT |
| TLR5 | CACAGTCACCAAACCAGGGA | GGGCAAAGTCAATTGCCAGG |
| TLR6 | GCAGGGGACAATCCATTCCA | AGAATCAGGCCAGCCCTCTA |
| TLR8 | AAACTTGACCCAACTTCGATACCTAA | GATCCAGCACCTTCAGATGAGG |
| TLR9 | TGCCCAAACTGGAAGTCCTC | TAAGGTTGAGCTCTCGCAGC |
| GAPDH | CGACAGTCAGCCGCATCTT | CCAATACGACCAAATCCGTTG |
| IL-1β | GGCTGCTCTGGGATTCTCTT | ATTTCACTGGCGAGCTCAGG |
| Rac1 | AAACCGGTGAATCTGGGCTT | TCGGCAATCGGCTTGTCTTT |
| PI3K | CATCACTTCCTCCTGCTCTAT | CAGTTGTTGGCAATCTTCTTC |
| Myd88 | GGACCCAGCATTGGGCATA | CGATGAGCTCACTAGCAATAGACC |
| p38 | TATGCGTCTGACAGGAACAC | GATCGGCCACTGGTTCATCA |
| JNK | GGAAAACGCTGACTCAGAACAC | CGGGTGTTGGAGAGCTTCAT |
| JUN | GGATCAAGGCGGAGAGGAGA | GGTTAGCATGAGTTGGCAC |
| Hp23srRNA | TAAGGCGCGTGAAAGAACTCTGGTTAA | TCGGGAGGGACTCTTTGCTGAGA |
| Hp23srRNA-probe | FAM-ACTCTGCAAACTAGCACCGTAAGTTCGCG-HEX |  |
| Gerbil-GAPDH | TTCAACGGCACAGTCAAGGC | GCCTTCTCCATGGTGGTGAAG |
| Gerbil-GAPDH-probe | FAM-CCC ATC ACC ATC TTC CAG GAG CGA GA-HEX |  |
|  |  |  |
